# Skin and Soft Tissue Infections (Patera Foot) in Immigrants, Spain

**DOI:** 10.3201/eid1504.081457

**Published:** 2009-04

**Authors:** Hugo-Guillermo Ternavasio-de la Vega, Alfonso Ángel-Moreno, Michele Hernández-Cabrera, Elena Pisos-Álamo, Margarita Bolaños-Rivero, Cristina Carranza-Rodriguez, Antonio Calderín-Ortega, José-Luis Pérez-Arellano

**Affiliations:** Hospital Universitario of Salamanca, Salamanca, Spain (H.-G. Ternavasio-de la Vega); Clinica Puerta de Hierro, Madrid, Spain (A. Ángel-Moreno); Hospital Universitario Insular of Gran Canaria, Gran Canaria, Spain (M. Hernández-Cabrera, E. Pisos-Álamo, M. Bolaños-Rivero, C. Carranza-Rodriguez, A. Calderín-Ortega, J.L. Pérez-Arellano); University of Las Palmas of Gran Canaria, Gran Canaria (M. Hernández-Cabrera, C. Carranza-Rodriguez, J.L. Pérez-Arellano)

**Keywords:** Skin and soft tissue infection, immigrant, patera foot, Shewanella algae, Spain, sub-Saharan Africa, dispatch

## Abstract

An unusual skin and soft tissue infection of the lower limbs has been observed in immigrants from sub-Saharan Africa who cross the Atlantic Ocean crowded on small fishing boats (*pateras*). Response to conventional treatment is usually poor. Extreme extrinsic factors (including new pathogens) may contribute to the etiology of the infection and its pathogenesis.

Immigration is increasing from poor-resourced countries into Spain and other European countries (*1*). From the coasts of Mauritania and Morocco, each year ≈10,000 African people try to reach the coasts of the Canary Islands or the southern Iberian Peninsula by sea, aboard small boats (called *pateras*) (*2*). These boats, normally used for fishing, have capacity for only a few persons but these sea crossings are overcrowded with 40–50 persons and with minimal water and food provisions. The journey lasts several days, during which travelers are exposed to extreme conditions, including cold weather; deficient hygiene; prolonged sitting in the same position; and prolonged immersion of their feet in sea water possibly contaminated by traces of feces, urine, decaying food, or fuel–water emulsions. On arrival, many need medical care for hypothermia and dehydration. However, despite efforts of authorities to prevent humanitarian disaster, frequently these boats sink, and bodies are later found along the coast of the Canary Islands.

Among the varied medical problems affecting these young, previously healthy immigrants are unexpectedly high numbers of severe skin and soft tissue infections (SSTIs), especially those involving the feet and legs. Clinical characteristics of these SSTIs were unfamiliar to our department, even though our unit collaborates directly with vascular surgeons, endocrinologists, and dermatologists to manage diabetes-related foot infections. The clinical picture comprises a painful cellulitis with minimal or imperceptible port of entry, deep abscesses, and tissue necrosis. Response to surgical debridement and broad-spectrum antimicrobial drugs is frequently poor, making amputation necessary in many cases. Here we describe the epidemiology, clinical features, microbiology, treatment, and outcome of 7 patients affected with severe SSTIs of the foot and leg and discuss the pathogenic role of *Shewanella algae* as an etiologic agent in this syndrome.

## The Study

The 7 patients were treated at our Unit of Infectious Diseases and Tropical Medicine at the Hospital Universitario Insular of Las Palmas (Gran Canaria Island, Canary Islands, Spain). We defined the condition we call “patera foot” as all of the following: 1) acute SSTI involving the feet or legs, 2) direct relation of the infection to sea journey by overcrowded *patera* under extreme conditions, and 3) good health status before travel. For this report, we excluded patients with diabetes, chronic arterial or venous leg disease, edema, or any other predisposing conditions.

We performed the following basic interventions for all patients: registry of epidemiologic data; complete clinical history and physical examination; blood extraction for routine tests; blood cultures (if fever); cleaning and debridement of the affected area (if indicated by the vascular surgery team); and acquisition of cultures from the affected area by syringe (if abscess), skin punch (if cellulitis only), or deep infected tissue (if surgical debridement). Plastic surgery was performed when indicated. Amputation was carried out only after every effort was made to preserve the affected foot or leg.

All 7 patients whose conditions met the classification criteria were young, black, sub-Saharan men (Table). In all patients, we ruled out a defined immunodeficiency, specifically HIV infection; use of immunosuppressive agents; and indirect data suggesting primary immunodeficiency (lymphopenia or immunoglobulin deficiency). Organisms isolated from local specimens were gram-negative bacteria in all microbiologically positive cases. Four patients underwent amputation, including 1 transtibial and 1 transmetatarsal.

**Table:** Clinical characteristics of 7 black male immigrants to Spain who developed severe skin and soft tissue infections (patera foot) following sea crossing from Africa on small boats (*pateras*)

Patient no.	Age, y	Country of origin	Isolated organism	Outcome
1	20	Ghana	Unknown	Transtibial amputation
2	38	Guinea	*Morganella morganii*	5th toe amputation
3	16	Mali	*Proteus vulgaris*	4th toe amputation
4	36	Guinea Bissau	*Enterobacter cloacae, Serratia* spp.	No amputation
5	27	Togo	Unknown	No amputation
6	20	Gambia	*Escherichia coli*	Transmetatarsal amputation
7	21	Côte d’Ivoire	*Shewanella algae*	No amputation; skin allograft

Patient 7, a 21-year-old man, was admitted to the intensive care unit because of shock secondary to severe SSTI and intense dehydration with acute renal failure and rhabdomyolysis. Bilateral painful enlargement of his lower extremities was evident, with disseminated round ulcers and serohemorrhagic, confluent blisters. The ulcers were covered with a fibrinoid, purulent exudate (Figure, panel A). After blood and skin samples were collected for culture, the patient began taking meropenem and linezolid . Skin samples grew gram-negative rods, initially identified as *S. putrefaciens* by using the API 20E system (bioMérieux, Marcy l’Etoile, France). By using experimental work (*3*–*5*) that allows differentiation between *Shewanella* species, we identified the organism as *S. algae* on the basis of its capacity to grow at 42ºC in 6.5% NaCl and to produce beta-hemolysis. The organism displayed in vitro susceptibility to ceftazidime, meropenem, piperacilin/tazobactam, ciprofloxacin, aminoglycosides, trimethoprim/sulfamethoxazole and aztreonam but resistance to amoxicilin/clavulamate, cefotaxime, and, notably, to imipenem (because *S. algae* is sensitive to other carbapenems i.e., meropenem). Four days after the patient was admitted to our unit, a large fluctuant area appeared on the dorsum of his left foot; drainage from the area consisted of a grossly purulent, foul-smelling material. Extensive debridement was necessary to control infection (Figure, panel B); 1 month later, a skin allograft was implanted (Figure, panel C). The patient was discharged, asymptomatic, after 70 days of hospitalization.

**Figure:**
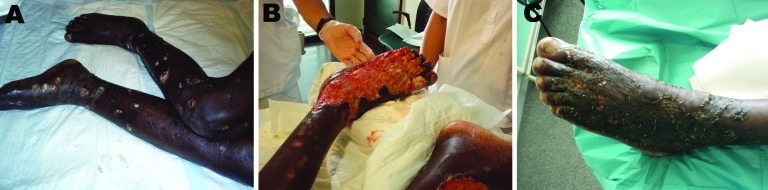
Progression of infection (patera foot) in case-patient 7, a previously healthy 21-year-old immigrant from sub-Saharan Africa who reached Spain by sea crossing on a small boat (*patera*). A) Initial severe skin and soft tissue infection of the lower limbs; B) extensive debridement of the left foot; C) left foot after skin allograft.

## Conclusions

During sea crossing by *patera*, immigrants are exposed to extreme extrinsic conditions, such as cold weather and deficient hygienic conditions. Intrinsic factors, such as limited skin compliance related to young age and possibly race, may play additional roles in the pathogenesis of this syndrome. Black race may be an intrinsic factor because, to our knowledge, immigrants of other ethnic origin (Magreb countries) have not developed this syndrome.

The presence of gram-negative bacteria in all case-patients, especially *S. algae* in 1, instead of gram-positive cocci, indicates a source of infection related to water and illustrates the specific pathogenesis of this syndrome. *Shewanella* spp*.* are ubiquitous gram-negative bacteria; possible reservoirs include all types of water, oil emulsions, petroleum brines, protein-rich foods, and soil (*5*–*7*). Two *Shewanella* species, *S. algae* and *S. putrefaciens*, have been found in clinical specimens. Because automated systems are unable to distinguish between the 2 species, a number of infections attributed to *S. putrefaciens* probably correspond to *S. algae* (*4*). *S. algae* is considered a rare opportunistic pathogen for humans, frequently involving immunocompromised hosts (*6*,*8*,*9*), and are usually part of a polymicrobial infection (*6*,*10*,*11*), which may mask its clinical importance. The presence of chronic leg ulcers in the context of peripheral vascular disease occurs commonly in adults with *S. algae* SSTI (*6*,*10*,*12*,*13*), and the affinity of *S. algae* for necrotic or ischemic tissues has been well described (*12*,*14*,*15*).

The following sequence may explain why these young, previously healthy men developed such aggressive SSTIs. We speculate that specific etiologic agents (mainly GNB, including *S. algae*) present in densely contaminated water enter through macerated skin, then reach deep tissues that have been submitted to subacute ischemia from overpressure and deficient venous drainage, both related to forced, prolonged sitting. The ensuing inner inflammation, expanding against a young skin with limited compliance, further aggravates the ischemia and leads to necrosis, probably by a compartmental-like mechanism.

These cases appear to represent a new syndrome, with specific etiology, pathogenesis, clinical features, and response to treatment. GNB, including *S. algae*, are involved, and an ischemic mechanism may be crucial in the development of these destructive infections. The initial election of empirical therapy, always covering those pathogens, and early surgical evaluation are crucial in preventing major disability in these young people.
